# Tracing fucosylation and astrocyte-associated pathogenic pattern in major depressive disorder: evidence from machine learning-based multi-omics and clinical validation

**DOI:** 10.3389/fnins.2026.1929767

**Published:** 2026-08-12

**Authors:** Yueting Kang, Wentao Sun, Zhibo Liu

**Affiliations:** 1 Division of Hypertension and Vascular Diseases, Department of Cardiology, Heart Center, The First Affiliated Hospital, Sun Yat-sen University, Guangzhou, Guangdong, China; 2 Department of Rheumatology, Beijing Hospital, Beijing, China; 3 Department of Anesthesiology, Air Force Characteristic Medical Center, Beijing, China

**Keywords:** astrocytes, fucosylation, G6PD, machine learning, major depressive disorder, multi-omics

## Abstract

**Background:**

Major depressive disorder (MDD) remains a debilitating psychiatric condition with substantial global prevalence. Emerging evidence highlights the role of aberrant fucosylation (FUS) and astrocyte dysfunction as key contributors to MDD pathogenesis; however, their reciprocal molecular interactions remain poorly defined.

**Objective:**

To identify and validate a fucosylation-astrocyte (FA)-associated molecular signature and its central pathogenic factor in MDD through a multi-omics and machine learning framework.

**Methods:**

We employed Limma, xCell, and WGCNA on bulk RNA-seq data from MDD patient brain tissues (GSE54566, GSE54570) to identify FA-associated shared differentially expressed genes (DEGs). Lasso-logistic regression was applied to the GSE53987 (MDD patient bulk training set) to identify the FAA-associated central pathogenic factor which molecular functions was estimated by single-gene GSEA analysis. The diagnostic performance of this gene was validated in GSE53987 and GSE241921 (MDD patient bulk brain independent set). Consensus clustering identified FA-associated molecular subgroups in GSE54564 (MDD patient bulk brain validation set). Single-cell data (GSE336230) of MDD brain patient tissues explored the cellular localization and pathogenic role of the hub gene in astrocytes. Drug screening was performed using the Drug Reflector platform in GSE53987, followed by molecular docking for enrichment therapeutic candidate targeting hub gene for MDD. Finally, clinical validation of hub gene expression was performed on MDD brain postmortem amygdala samples from 10 MDD patients and 10 controls using q-RT-PCR.

**Results:**

We identified 8 FA-associated shared DEGs, which can stratify MDD patient into 2 molecular groups. G6PD can be considered as the up-regulated potential diagnostic pathogenic factor, which was mainly distributed in astrocyte. BRD-K97481123 can be a potential therapeutic candidate with G6PD.

**Conclusion:**

Our findings unveil a novel FA-associated pathogenic signature can be considered as a potential therapeutic target for MDD. This study provides a new framework for understanding MDD to enhance clinical translation.

## 1 Introduction

Major depressive disorder (MDD) is a principal cause of disability globally, affecting over 280 million individuals and contributing substantially to the worldwide disease burden (Belmaker and Agam, 2008). Its pathophysiology is multifaceted, involving dysregulation of monoaminergic neurotransmission, hypothalamic–pituitary–adrenal (HPA) axis, neuroinflammation, and impaired neuroplasticity (Johnson et al., 2025). Despite decades of research, the diagnostic process remains entirely clinical, and currently available antidepressants exhibit limited efficacy and a substantial lag in therapeutic onset, highlighting an urgent need for biomarker-driven diagnostics and mechanistically novel therapeutic targets (Schechter, 2005). The underlying molecular heterogeneity of MDD suggests that distinct biological pathways contribute to its pathogenesis in different patient subgroups.

Recent investigations have shifted focus toward post-translational modifications and glial cell biology in MDD (Wu M. S. et al., 2022). Fucosylation, a specific type of glycosylation involving the addition of fucose sugar residues to proteins, is critical for cell–cell adhesion, signal transduction, and immune modulation (Boeck et al., 2018). Dysregulated fucosylation (FUS) has been implicated in neurodevelopmental and neurodegenerative disorders, yet its role in MDD remains obscure (Park et al., 2018). Concurrently, astrocytes, the most abundant glial cell type in the central nervous system, are increasingly recognized for their essential roles beyond metabolic support (Fulton et al., 2025). Reactive astrogliosis and altered astrocyte function, including disrupted glutamate homeostasis and impaired neurotrophic factor release, are now considered hallmarks of MDD pathophysiology (Fulton et al., 2025). However, the molecular crosstalk between abnormal FUS and astrocyte dysfunction in the context of MDD has not been systematically investigated (Jiao et al., 2025).

To address this gap, we employed an integrative multi-omics strategy utilizing human brain transcriptomic data in conjunction with an interpretable machine learning pipeline. Our objective was to decode a fucosylation–astrocyte (FA)-associated molecular signature in MDD, with the goal of identifying a shared molecular nexus between these processes and pinpointing a central pathogenic driver and therapeutic target for MDD patients.

## 2 Materials and methods

### 2.1 Transcriptomic data acquisition

Bulk RNA-sequencing datasets derived from postmortem brain tissue of MDD patients and healthy controls were retrieved from the GEO database via GEOquery package in R (Tu et al., 2023). GSE54566 comprised 14 MDD and 14 control samples (Chang et al., 2014). GSE54570 included 13 MDD and 13 control samples (Su et al., 2024). GSE53987 contained 55 MDD and 50 control samples (Chen et al., 2024). GSE54564 contained 21 MDD and 21 control samples (Chang et al., 2014). GSE54565 contained 21 MDD (Chang et al., 2014). Raw data were normalized using the limma package in R (Ritchie et al., 2015). A curated list of FUS-related genes was compiled from the GeneCards database using a relevance score threshold greater than 1.

### 2.2 Differential expression and functional enrichment

To delineate the transcriptomic landscape associated with MDD, differential expression analysis was executed between the MDD and control groups within GSE54566 via limma (Ritchie et al., 2015). Transcripts meeting an absolute log₂ fold change (FC) threshold of 0.5 alongside a *p*-value < 0.05 were designated as differentially expressed genes (DEGs) (Ritchie et al., 2015). These identified DEGs were subsequently overlapped with the FUS-related gene list to isolate FUS-associated candidates (Xu et al., 2023). Heatmap visualizations of expression profiles were generated using pheatmap of which biological pathway enrichment, specifically covering GO terms and KEGG pathways, was conducted through the clusterProfiler package, leveraging the underlying gene sets from the MSIGDB repository (Xu et al., 2023).

### 2.3 Astrocyte abundance of expression and co-expression network analysis

We quantified the relative infiltration of astrocytes within the GSE54570 brain microenvironment by deploying the xCell algorithm (Zhang et al., 2025). To elucidate gene modules correlated with these astrocytic proportions, WGCNA was implemented via the corresponding R package (Langfelder and Horvath, 2008). A suitable soft-thresholding power (*β*) was iteratively selected to meet the scale-free topology criterion (R^2^ > 0.9) (Langfelder and Horvath, 2008). Following the derivation of a TOM, hierarchical clustering was performed with a minimum module size of 60 genes (Langfelder and Horvath, 2008). The module whose eigengene displayed the most robust positive linkage to the astrocyte score was retained as the astrocyte-associated module (Langfelder and Horvath, 2008). Genes within this module were then intersected with the FUS-associated DEGs, yielding the final set of FA-associated shared DEGs. DO annotation was carried out via the DOSE package, and chromosomal mapping of these genes was visualized by means of circlize package in R (Chen et al., 2026).

### 2.4 Machine learning analysis and diagnostic model construction

With the goal of developing a sparse diagnostic classifier, Lasso-logistic regression was trained on the GSE53987 dataset using the caret package in R (Tang et al., 2021). The regularized model parameters were optimized through a 10-fold cross-validation scheme repeated five times, with the optimal *λ* (lambda.min) guiding feature selection (Tang et al., 2021). The predictive performance of the resulting model was then stringently evaluated in both the training set (GSE53987) and an independent validation cohort (GSE241921) through ROC and PR analyses generated by the pROC library (Robin et al., 2011). The calibration accuracy of the model was examined via calibration plots, and a practical nomogram was subsequently constructed with the rms package to illustrate individualized risk prediction (Lin et al., 2024).

### 2.5 Consensus clustering for molecular subtyping

To explore intrinsic biological subgroups within the MDD population, consensus clustering was applied to the GSE54564 cohort based on the expression signatures of the previously identified FA-associated shared DEGs. This process was executed via the ConsensusClusterPlus package (Hu et al., 2022). The optimal cluster count was established at *k* = 2 by systematically inspecting the consensus matrix heatmap, the cumulative distribution function (CDF) trajectories, and delta area plots (Hu et al., 2022). To characterize the biological disparities between these emerging subtypes, we employed the CIBERSORT algorithm to compare immune cell infiltration landscapes and performed GSEA via clusterProfiler to identify pathway-level differences, drawing upon KEGG and GO references from MSIGDB (Zhou et al., 2026).

### 2.6 Single-cell RNA sequencing analysis

The single-cell transcriptomic dataset (GSE336230, comprising 9 MDD brain tissue samples) was processed and analyzed using the Seurat pipeline in R (Butler et al., 2018). Stringent quality control steps were applied, filtering out cells with fewer than 200 or more than 6,000 unique feature counts, or those exhibiting a mitochondrial gene ratio exceeding 15% (Butler et al., 2018). Following normalization via the SCTransform method, the top 2,000 highly variable genes were extracted for downstream analysis (Butler et al., 2018). Principal component analysis (PCA) was performed, followed by graph-based clustering (resolution = 0.6), and the resultant clusters were visualized using UMAP and *t*-SNE plots (Butler et al., 2018). Cell-type annotations were assigned by the SingleR package, referencing the Human Primary Cell Atlas (Cao et al., 2023). Intercellular communication networks were mapped using CellChat, while metabolic heterogeneity across cell states was explored via scMetabolism (Jin et al., 2025; Wu Y. et al., 2022). To quantify the activity of FUS-related pathways in distinct cell populations, we applied the AUCell scoring algorithm (Ma et al., 2024). To probe the functional consequences of our hub gene within astrocytes, we conducted an in silico knockout simulation using scTenifoldKnk (Osorio et al., 2022). The perturbed gene profiles were subjected to functional enrichment analysis and GENEMINIA via clusterProfiler to map the affected molecular pathways (Wang et al., 2026). Additionally, the pseudotime developmental trajectories of astrocytes, along with the dynamic expression of the targeted gene, were reconstructed with the Monocle2 framework (Huang et al., 2024).

### 2.7 Drug screening and molecular docking analysis

To identify potential therapeutic agents, the AI-driven DrugReflector platform was employed for drug repurposing screening (Ma, 2026). The bulk expression signature derived from the GSE53987 cohort was queried against a library of small bioactive molecules compiled from the cMAP repository (Ma, 2026). Compounds exhibiting a predicted inverse correlation with the disease profile were ranked according to a drug-target association score (DTS) exceeding 0.9 (Ma, 2026). The highest-ranked candidate, BRD-K97481123, was subsequently advanced to molecular docking studies (Ma, 2026). The three-dimensional crystal structure of the target protein was retrieved from the RCSB PDB database, while the molecular configuration of BRD-K97481123 was obtained from the PubChem database (Ma, 2026). Docking simulations were carried out using AutoDock Vina, and the most energetically favorable binding poses were visualized with the PyMOL software (Ma, 2026).

### 2.8 Clinical validation

Postmortem amygdala specimens were procured from a study cohort consisting of 10 MDD patients and 10 age-matched neurotypical controls, following institutional ethical approval and the acquisition of signed donor consent forms. For total RNA recovery, TRIzol™ reagent (Invitrogen, United States) was employed in strict accordance with the prescribed manufacturer’s protocol. Subsequently, 1 μg of the isolated RNA served as the template for first-strand cDNA synthesis, which was performed with the PrimeScript™ RT kit supplemented with gDNA Eraser (Takara Bio, Japan). Quantitative real-time PCR reactions were then executed on a QuantStudio™ 5 platform (Applied Biosystems, United States), using TB Green® Premix Ex Taq™ II (Takara Bio, Japan) as the detection agent. The complete sequences of the oligonucleotide primers designed for target gene amplification are detailed below:

G6PD:

F: 5‘-AGGACCAGGACCTCTTTAGC-3’,

R: 5‘-TGCAGCACCTTGACATTTCC-3’;

GAPDH:

F: 5‘-GGAGCGAGATCCCTCCAAAAT-3’,

R: 5‘-GGCTGTTGTCATACTTCTCATGG-3’.

### 2.9 Statistical analysis

All statistical analyses were executed using R (version 4.2.2) or GraphPad Prism (version 9.2.2). Between-group comparisons were performed using Student’s t-test for normally distributed data or the Mann–Whitney U test for non-normal distributions. Multiple group comparisons were analyzed via one-way ANOVA followed by Tukey’s post-hoc test. Correlations were assessed using Pearson’s or Spearman’s tests as appropriate. A *p*-value < 0.05 or an FDR < 0.05 was deemed statistically significant.

## 3 Results

### 3.1 Identification of FUS-associated molecular patterns for MDD patients

**Figure 1:**
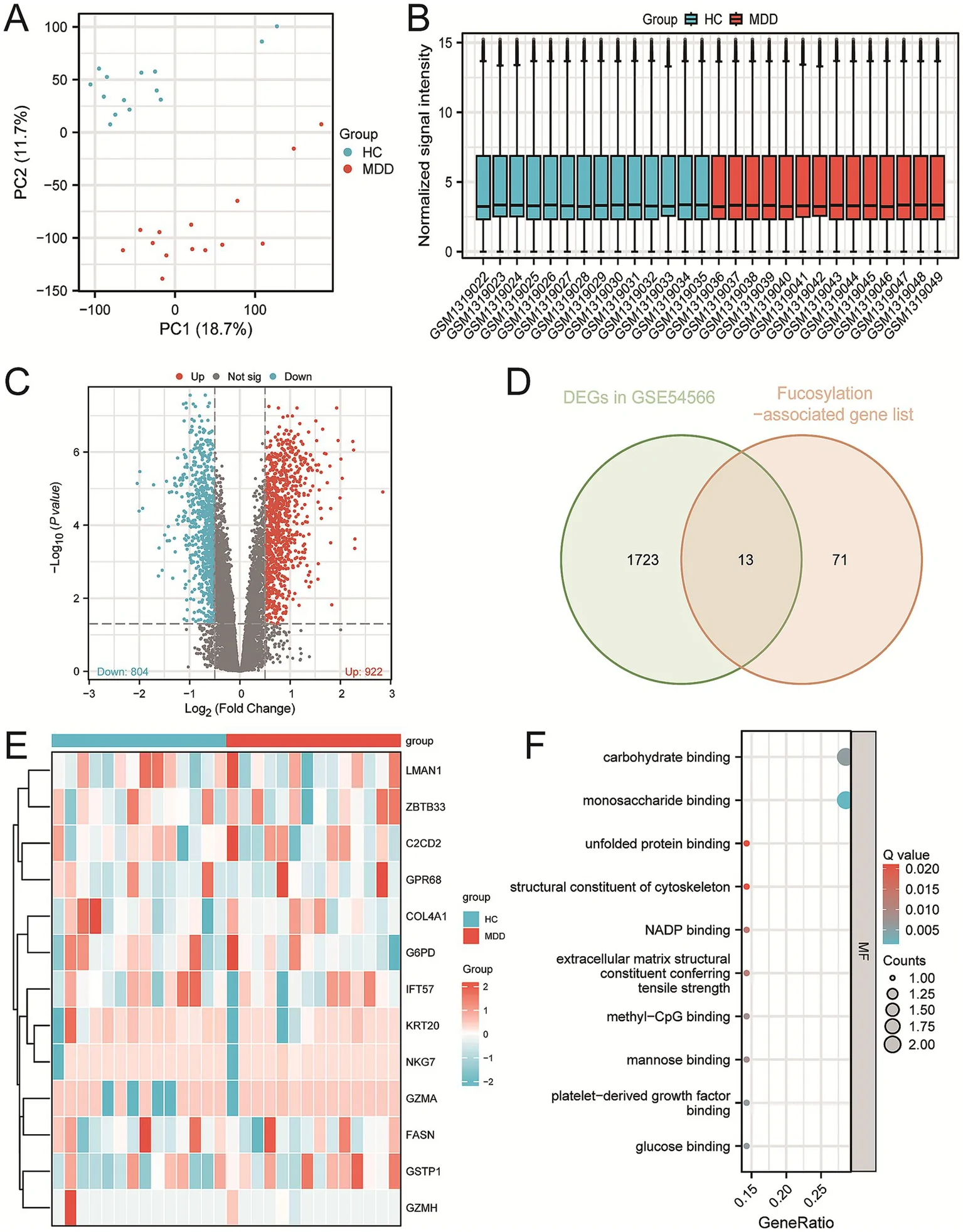
Identification of FUS-associated DEGs in MDD. **(A)** PCA plot of normalization results of GSE54566. **(B)** Box plot of normalization results of GSE54566. **(C)** Volcano plot of DEGs in GSE54566. **(D)** Venn diagram depicting the intersection of DEGs from GSE54566 and fucosylation-related genes. **(E)** Heatmap showing expression patterns of 13 FUS-associated DEGs. **(F)** GO enrichment analysis of these 13 DEGs. Data with a threshold of *p* or FDR < 0.05 were considered statistically significant.

Differential analysis of the GSE54566 dataset revealed 922 upregulated and 804 downregulated DEGs between MDD and control groups (Figures 1A–C). Upon intersecting these DEGs with the curated fucosylation-related gene set, 13 FUS-associated DEGs were identified (Figure 1D). A heatmap visualized the distinct separation of these 13 genes between MDD and control tissues (Figure 1E). Functional enrichment analyses indicated that these genes were significantly implicated in NADP metabolic processes and carbohydrate metabolism (Figure 1F).

### 3.2 Estimation of FA-associated molecular patterns for MDD patients

**Figure 2:**
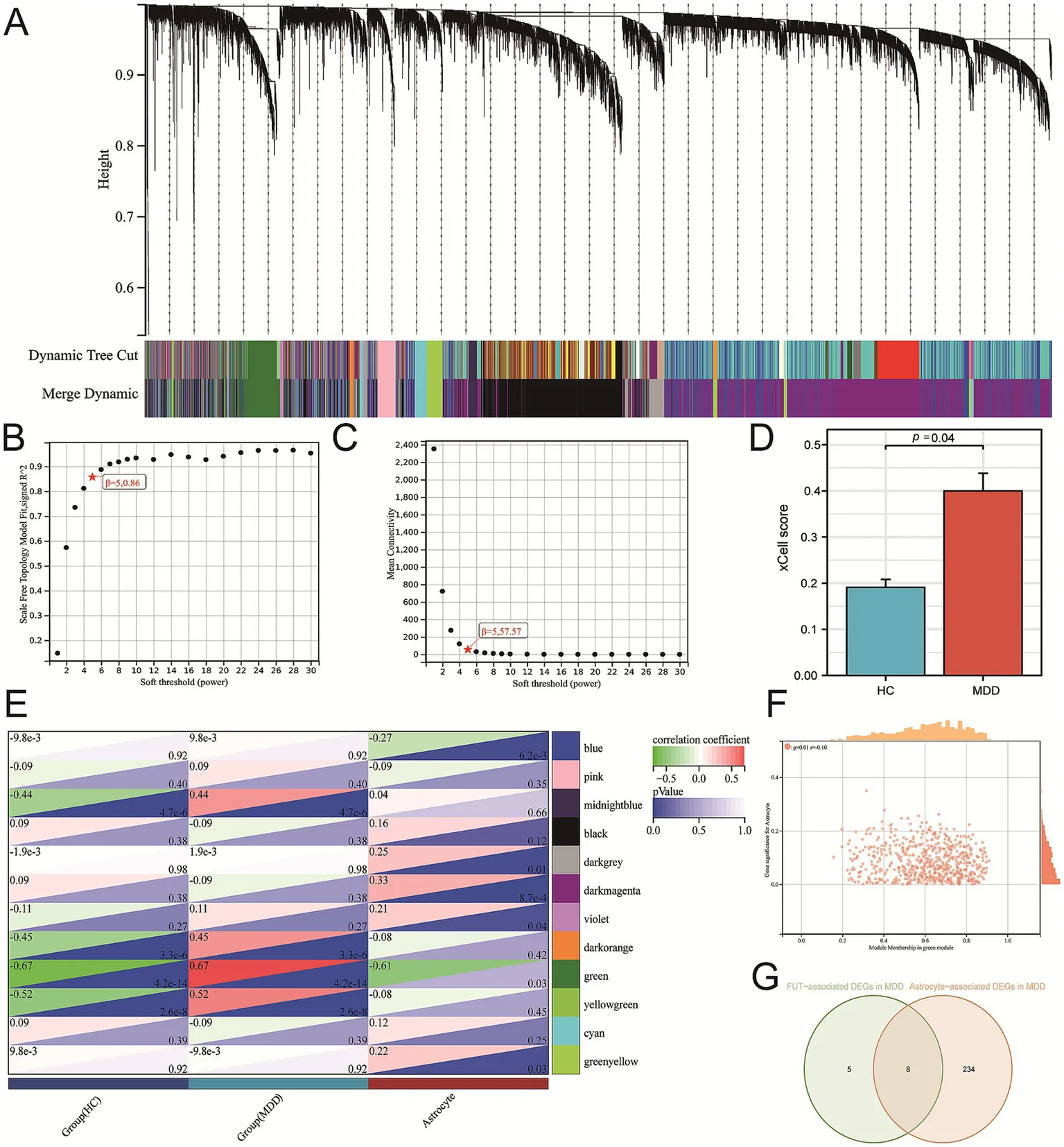
Identification of FA-associated shared DEGs in MDD. **(A–C)** Dendrogram of WGCNA modules. **(D)** Box plot showing increased astrocyte infiltration in MDD samples. **(E,F)** Module-trait correlations with xCell analysis astrocyte score. **(G)** Venn diagram showing the 8-gene FA-associated gene signature. Data with a threshold of *p* < 0.05 were considered statistically significant.

Using the xCell algorithm on GSE54566, we observed a significantly higher proportion of astrocytes in the MDD brain microenvironment compared to controls (Figure 2D). After identification of optimal threshold, WGCNA identified 12 co-expression modules (Figures 2A–C). The Green module showed the strongest positive correlation with astrocyte abundance and was designated the astrocyte-associated module (Figures 2E,F). Intersecting the 242 genes from this module with the 13 FUS-associated DEGs yielded 8 FA-associated shared DEGs, including IFT57, G6PD, ZBTB33, C2CD2, LMAN1, KRT20, GPR68, and COL4A1 (Figure 2G).

### 3.3 Identification of FA-associated diagnostic signature for MDD patients

**Figure 3:**
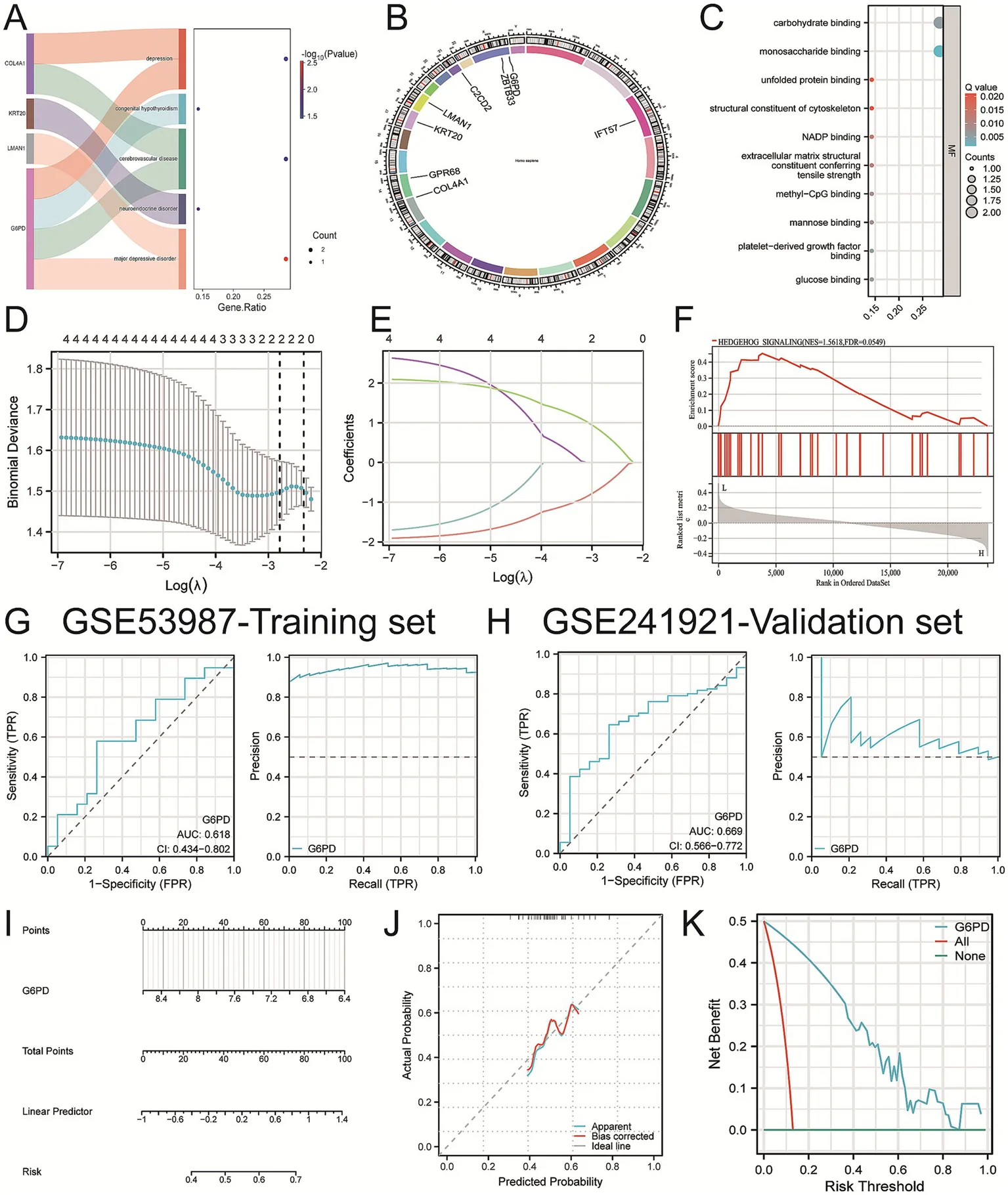
Identification and validation of G6PD as a diagnostic signature in MDD. **(A)** DO analysis of FA-related DEGs. **(B)** Chromatin localization of FA-related DEGs. **(C)** KEGG and GO analysis of FA-related DEGs. **(D,E)** Lasso-logistic regression analysis. **(F)** Single-gene GSEA analysis of G6PD in GSE53987. **(G)** ROC and PR analysis of G6PD in GSE53987. **(H)** ROC and PR analysis of G6PD in GSE241921. **(I–K)** Nomogram and calibration curve in GSE53987. Data with a threshold of *p* or FDR < 0.05 were considered statistically significant.

DO analysis identified that LMAN1 and G6PD were 2 MDD-associated pathogenic factors among FA-associated DEGs (Figure 3A). Next, for FA-associated DEGs, we analyzed molecular functions and chromatin localization (Figures 3B,C). Next, Lasso-logistic regression in GSE53987 identified G6PD as a FA-associated hub gene which positively regulates Hedgehog signaling (Figures 3D,F). This diagnostic accuracy was validated in GSE53987 (AUC = 0.618) and GSE241921 (AUC = 0.669) (Figures 3G,H). The nomogram and calibration curve indicated potential agreement between predicted and observed probabilities (Figures 3I,K).

### 3.4 FA-associated molecular subgroup identification for MDD patients

**Figure 4:**
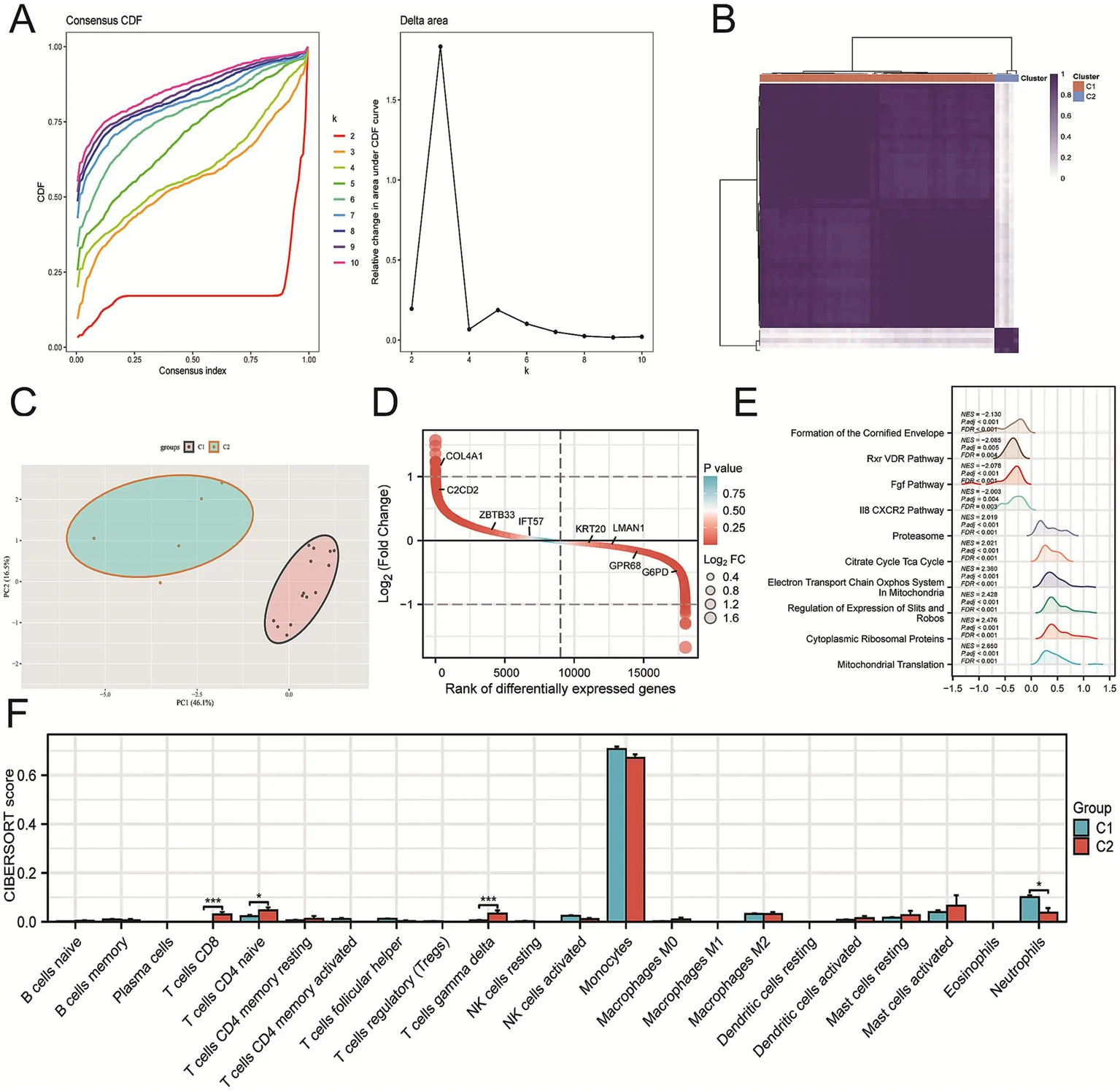
FA-associated molecular subtyping in MDD. **(A,B)** Consensus matrix heatmap for *k* = 2 and corresponding CDF curve and delta area plot. **(C)** PCA analysis of subtypes. **(D)** Heatmap of 8 FA-related signature genes across subtypes. **(E)** GSEA ridge plot displaying enriched pathways between the two subgroups, with NES and FDR values indicated. **(F)** GSEA results of different subgroups. Data with a threshold of *p* or FDR < 0.05 and were considered as significant.

Consensus clustering, based on the 8-gene FA signature, stratified MDD patients in GSE54564 into two distinct subgroups, designated C1 and C2 (Figures 4A–C). The differential expression patterns of all 8 signature genes were assessed (Figure 4D). GSEA revealed that the C1 subtype was enriched in inflammatory pathways, whereas the C2 subtype was dominated by metabolic processes (Figure 4E). CIBERSORT analysis revealed starkly different immune microenvironments: the C2 cluster exhibited higher infiltration of CD8 + T cells, CD4 + naïve T cells, and gamma delta T cells, while the C1 cluster featured a higher proportion of neutrophils (Figure 4F).

### 3.5 Identification of astrocyte patterns in MDD microenvironment

**Figure 5:**
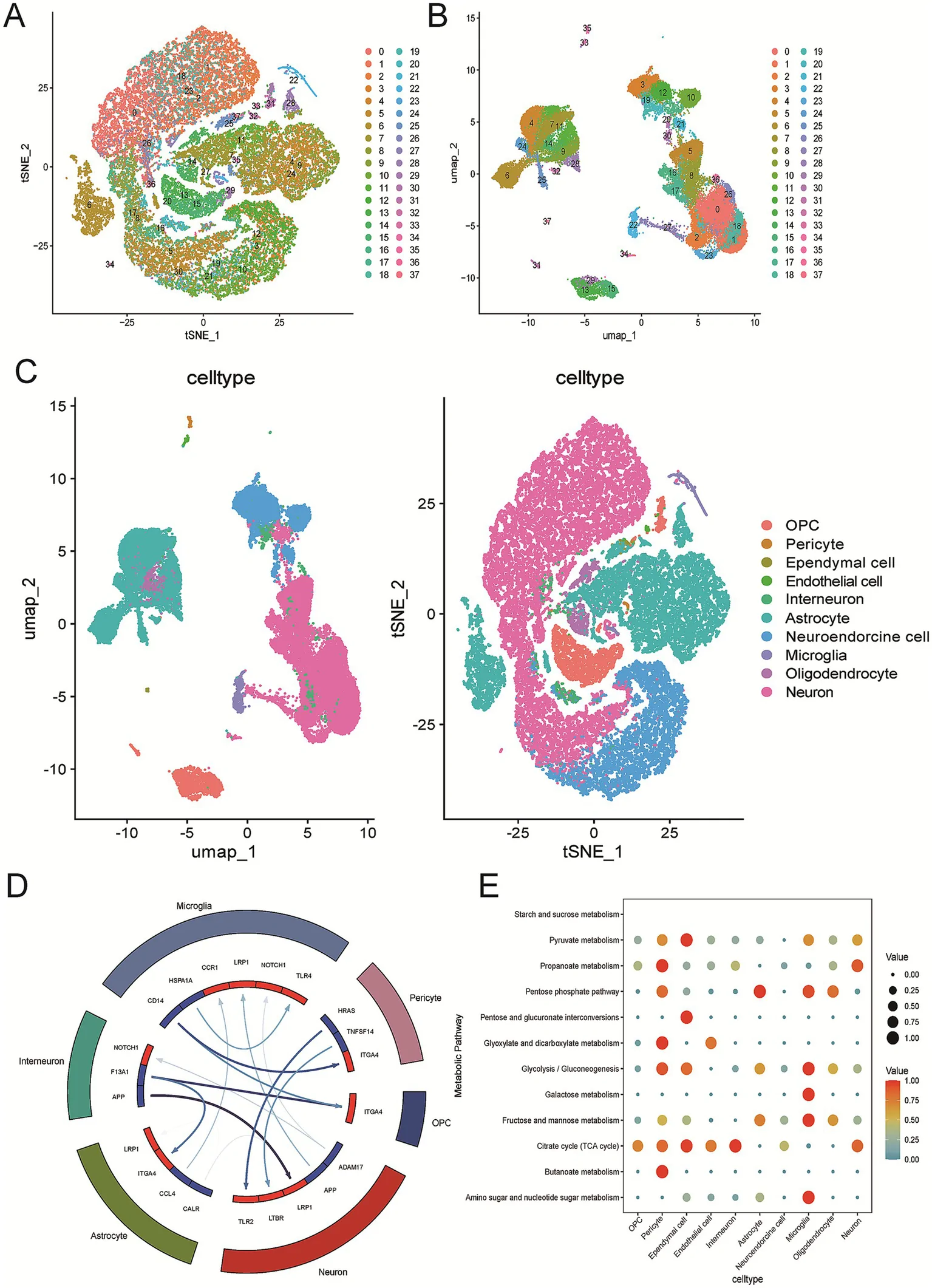
Single-cell landscape of astrocyte in MDD brain. **(A,B)** UMAP and *t*-SNE plot of 38 cell clusters. **(C)** UMAP and *t*-SNE plot of annotated cell type. **(D)** Cell–cell communication heatmap. **(E)** Metabolic pathway activity in astrocytes. Data with a threshold of *p* or FDR < 0.05 were considered statistically significant.

Single-cell analysis of GSE336230 identified 37 distinct cell clusters after quality control and dimensionality reduction (Figures 5A,B). Astrocytes were identified based on SingleR annotation (Figure 5C). Cell–cell communication analysis revealed that astrocytes predominantly interacted with interneurons, notably through the F3A1-ITGA4 ligand-receptor pair (Figure 5D). Metabolic heterogeneity analysis showed that the pentose phosphate pathway (PPP) was specifically activated in astrocytes (Figure 5E).

### 3.6 Mechanisms of FA-associated central pathogenic factor in astrocyte for MDD

**Figure 6:**
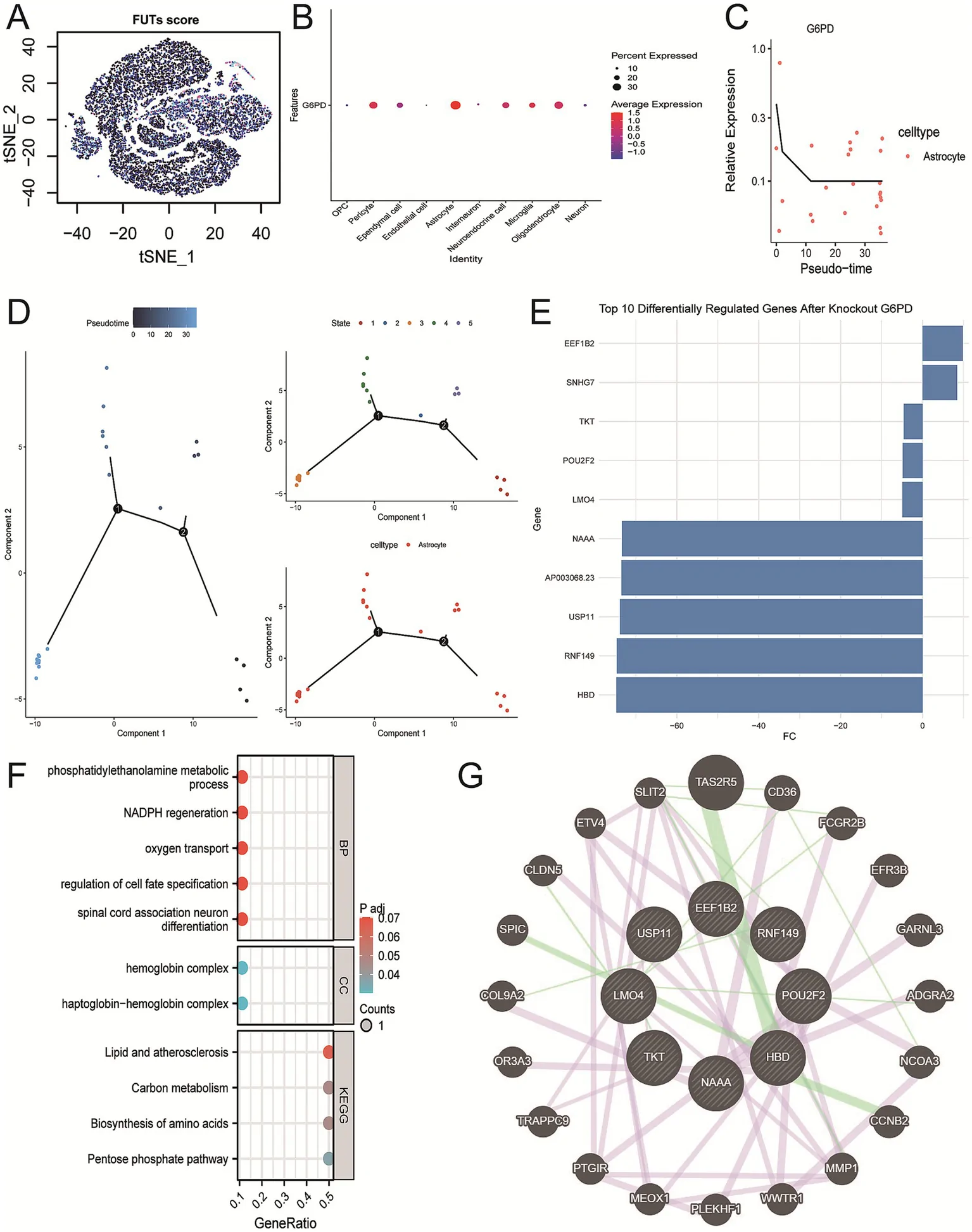
G6PD as a central pathogenic factor in astrocytes of MDD. **(A)** AUCell scores for fucosylation pathway across cell types. **(B)** Feature plot of G6PD expression. **(C,D)** Pseudotime trajectory of astrocytes and G6PD expression along pseudotime. **(E–G)** Virtual KO of G6PD in astrocyte and corresponding molecular function enrichment. Data with a threshold of *p* or FDR < 0.05 were considered statistically significant.

AUCell analysis demonstrated significant activation of the fucosylation pathway specifically within the astrocyte cluster (Figure 6A). G6PD was found to be predominantly expressed in astrocytes and was subsequently designated the central pathogenic driver (Figure 6B). Pseudotime trajectory analysis of astrocytes revealed five distinct differentiation branches, with G6PD expression showing progressive upregulation along these developmental paths (Figures 6C–D). Virtual knockout of G6PD in astrocytes, followed by enrichment analysis, revealed that the perturbed genes were potentially enriched in NADPH metabolism and epigenetic modification pathways (Figures 6E–G).

### 3.7 FA-associated hub molecular signature-oriented therapeutic candidate identification for MDD patients

**Figure 7:**
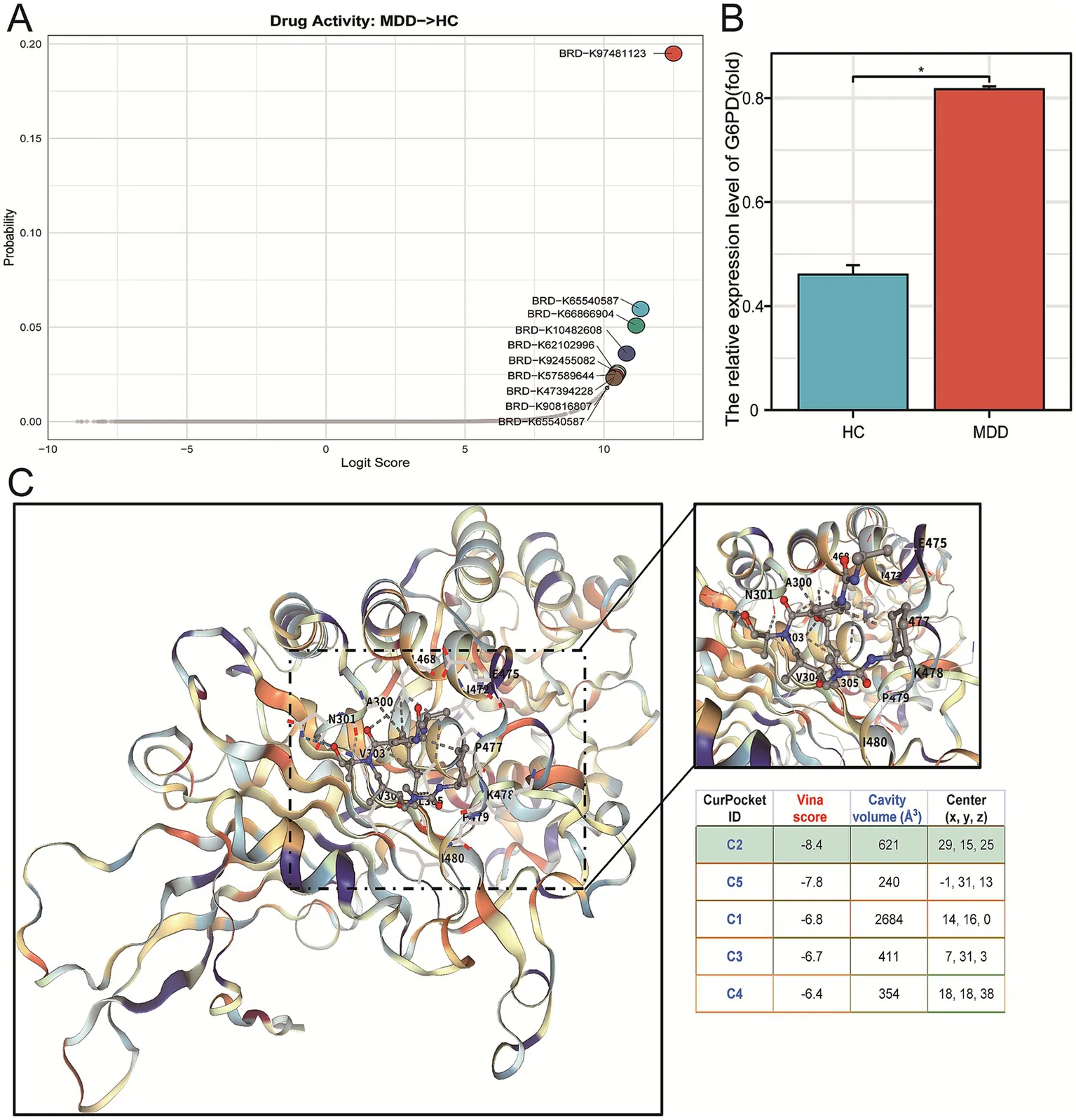
Drug screening and clinical validation targeting G6PD for MDD. **(A)** DrugReflector analysis based on GSE53987. **(B)** Q-RT-PCR validation of G6PD in the amygdala of MDD patients and controls. **(C)** Molecular docking pose of BRD-K97481123 with G6PD. Data with a threshold of *p* or FDR < 0.05 were considered statistically significant.

The DrugReflector platform nominated BRD-K97481123 as the top candidate compound, predicted to reverse the MDD-like expression profile in GSE53987 towards a control-like state (DTS = 0.95, Figure 7A). Molecular docking simulations confirmed a strong predicted binding affinity between BRD-K97481123 and G6PD, with a docking score of −8.4 kcal/mol, indicating a stable interaction (Figure 7C). Finally, qRT-PCR on postmortem amygdala tissues confirmed a significantly elevated G6PD mRNA level in MDD patients compared to healthy controls (Figure 7B).

## 4 Discussion and conclusion

In this study, we present a comprehensive multi-omics analysis that systematically deciphers a novel molecular signature linking FA-associated patterns in MDD. By integrating bulk transcriptomics, machine learning, single-cell analysis, and clinical validation, we identified G6PD as a core astrocyte-distributed pathogenic factor. Our findings not only provide a new diagnostic signature but also offer a mechanistic framework for understanding the role of glial-glycosylation crosstalk in MDD.

G6PD is the rate-limiting enzyme of the pentose phosphate pathway (PPP), which is crucial for generating NADPH and ribose-5-phosphate (Meng et al., 2022). NADPH is essential for maintaining the cellular redox balance by regenerating reduced glutathione, a key antioxidant (Greaney et al., 2019). In the context of MDD, chronic stress and neuroinflammation are known to induce oxidative stress (Ibi et al., 2017). Prior studies have reported altered G6PD activity in the brains of depressed patients, suggesting a link between impaired PPP flux and vulnerability to oxidative damage (Puthumana and Regenold, 2019). Our data aligns with this, demonstrating that G6PD is not only a reliable diagnostic marker but also that its dysregulation in astrocytes may lead to a compromised antioxidant capacity, contributing to the neurodegenerative and neuro-inflammatory environment characteristic of MDD. Furthermore, we discovered that G6PD positively regulates the Hedgehog signaling pathway in astrocytes. The Hedgehog pathway is critical for adult neurogenesis and glial cell function, and its dysregulation has been linked to depressive-like behaviors (Yu et al., 2021). This novel connection suggests that G6PD may act as a metabolic hub, integrating cellular energy metabolism, redox state, and developmental signaling pathways in astrocytes to influence mood regulation.

Our study identified a novel FA-associated molecular co-expression patterns in MDD, with G6PD pinpointed as a potential diagnostic biomarker and therapeutic target. The integration of multi-omics and machine learning provides a powerful framework for dissecting the molecular heterogeneity of MDD. By highlighting the role of G6PD in astrocyte metabolism and signaling, this work opens new avenues for developing glial-specific and metabolically targeted therapies for depression. However, there are still limitations of this study. The foundational transcriptomic insights are derived from publicly available datasets, which, despite batch correction caused by agonal state, medication effects, and RNA degradation, may not fully account for all sources of technical and biological variability. Future studies should combine the clinical information of MDD patients, focusing on the estimation of efficacy of diagnostic and molecular stratification models acquired from our study in a large, multi-center clinical trials to enhance clinical significance. Besides, The single-cell analysis, while offering high-resolution insights, is based on a cohort of 9 MDD patients, potentially limiting the capture of the full heterogeneity of astrocyte states across the AD spectrum. Future studies should combined advanced AI technologies in higher resolution single-cell and spatial data of MDD patients, coupled with pre-clinical studies, dynamically tracing FUS in astrocyte for the pathogenesis of MDD patients. In addition, the precise molecular cascades downstream of G6PD and FUS-modified G6PD in astrocytes that ultimately impair neuronal health remain to be fully delineated in pre-clinical MDD models. Furthermore, Future research should prioritize *in vivo* studies using conditional knockout or astrocyte-specific overexpression of G6PD in MDD mouse models to establish causality and elucidate detailed mechanisms acquired by our *in silico* knockout. Significantly, there are 10 MDD patient cerebral tissue samples and 10 HC samples were collected in our study, it may cause batch effects. Future study should focus on adopting multi-center MDD patient cerebral tissue samples for examination G6PD FUS-modified and Pan-FUS-modified mechanisms in MDD, offering novel idea in clinical translation. Similarly, the therapeutic candidate, BRD-K97481123, identified computationally, demands rigorous experimental testing in preclinical models to confirm its efficacy, optimal dosing, and ability to penetrate the blood–brain barrier and modulate the proposed FUS mechanism in astrocyte of MDD.

## Data Availability

All bulk and single-cell RNA-seq datasets analyzed in this study are publicly available from the Gene Expression Omnibus (GEO) database (https://www.ncbi.nlm.nih.gov/geo/) under accession numbers GSE54566, GSE54570, GSE53987, GSE54564, GSE241921 and GSE336230. The raw qRT-PCR data generated in this work are available from the corresponding author upon reasonable request.
